# Interfacial Fluorinated Ion Crowding Enables Reversible Zinc Metal Batteries

**DOI:** 10.1002/anie.3251724

**Published:** 2026-07-20

**Authors:** Xinyu Zhang, Jitao Shang, Ruwei Chen, Jianrui Feng, Hang Yang, Jingyi Wang, Fei Guo, Shuhui Li, Zijuan Du, Peie Jiang, Xiaoxia Guo, Wei Zhang, Jie Chen, Hongzhen He, Xuan Gao, Zhenjing Jiang, Bing Wang, Yuhang Dai, Guanjie He

**Affiliations:** ^1^ Christopher Ingold Laboratory Department of Chemistry University College London London UK; ^2^ Institute of Technological Sciences Wuhan University Wuhan China; ^3^ State Key Laboratory of Silicate Materials For Architectures Wuhan University of Technology Wuhan China; ^4^ Department of Engineering Science University of Oxford Oxford UK; ^5^ The Electrochemical Innovation Lab Department of Chemical Engineering University College London London UK; ^6^ SEU‐FEI Nano‐Pico Center Key Laboratory of MEMS of Ministry of Education Southeast University Nanjing China; ^7^ State Key Laboratory of Advanced Technology For Materials Synthesis and Processing Wuhan University of Technology Wuhan China

**Keywords:** aqueous solution, chemical engineering, electrochemistry, electrolyte, energy storage, molecule, nanotechnology, plating, zinc

## Abstract

Aqueous zinc metal batteries (AZMBs) are promising candidates for large‐scale energy storage owing to their intrinsic safety. However, their lifespan is severely limited by side reactions such as dendrite growth and hydrogen evolution at the Zn‐electrolyte interface. Conventional single‐electrolyte‐additive approaches are thermodynamically constrained, yielding only insufficient coverage of the inner‐Helmholtz plane (IHP) and poor control of interfacial reactions. Here, we report an interfacial fluorinated‐ion crowding strategy by simultaneously introducing multiple low‐concentration fluorinated additives. Computational and spectroscopic analyses reveal that various‐sized F‐groups densely occupy the IHP, displacing water molecules and homogenizing Zn^2+^ flux. This emergent crowding effect, inaccessible to single‐additive strategies, enables unprecedented interfacial regulation. Electrochemical tests demonstrate ultrastable Zn plating/stripping over 1200 h at 5 mA cm^−2^ and 1800 h at 10 mA cm^−2^, more than tenfold longer than the baseline electrolyte. This work establishes interfacial ion crowding as a powerful design principle, rooted in fundamental electrochemistry, offering a pathway toward high‐performance and durable AZMBs.

## Graphical abstract

Unlike conventional single‐anion electrolytes with limited interfacial regulation, interfacial F‐ion crowding enables dense occupation of the inner Helmholtz plane through cooperative fluorinated anions, thereby generating a fluorine‐rich interface that excludes water, homogenizes Zn^2+^ nucleation, promotes Zn (100)‐oriented deposition, and enables remarkably stable Zn metal anodes.

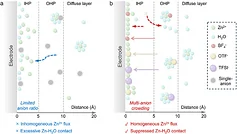

## 1 Introduction

Aqueous zinc metal batteries (AZMBs) are garnering significant global attention for large‐scale energy storage due to the abundance, low cost, and safety of zinc, making them one of the most promising next‐generation battery technologies [1, 2, 3]. However, their practical application is hindered by severe interfacial issues, including uncontrolled dendrite growth and parasitic hydrogen evolution reaction (HER) at the Zn‐electrolyte interface [4]. Considerable efforts have been devoted to bulk‐electrolyte engineering, such as high‐concentration “water‐in‐salt” and molecular crowding strategies, which suppress water activity and improve cycling stability [5, 6, 7, 8, 9]. Yet, these approaches inevitably compromise the intrinsic merits of aqueous systems by reducing ionic conductivity, sacrificing non‐flammability, introducing impurities, and causing ion aggregation or inhomogeneous ionic clusters, in addition to their high cost [10, 11, 12, 13].

Recent studies have turned to electrolyte molecular design as an alternative route to stabilize Zn interfaces. Strategies such as stereochemical engineering, synergistic cation–anion regulation, and pre‐solvation/encapsulation have shown the ability to tune Zn^2^^+^ hydration [9, 14, 15, 16]. Nevertheless, single‐species additives remain fundamentally constrained by competitive adsorption within the inner Helmholtz plane (IHP).

Given these limitations, interfacial engineering emerges as a more effective route to regulate Zn deposition and suppress HER. Current studies mainly focus on two directions. One is the construction of protective coatings [17] on Zn anodes, which can block parasitic reactions and stabilize cycling, although such films are prone to cracking and may hinder Zn^2+^ transport [15]. The other is the use of electrolyte additives to tune Zn nucleation and interfacial chemistry [18].

For electrolyte additives, traditional single‐ion species such as Zn(OTF)_2_, Zn(BF_4_)_2_, and Zn(TFSI)_2_ can function as solvation sheath modulators or solid‐electrolyte interphase (SEI) promoters [19, 20, 21]. However, their effectiveness is fundamentally constrained by double‐layer theory, since the surface concentration of a single additive in the inner Helmholtz plane (IHP) is inherently limited by competitive adsorption equilibria [19, 21]. The strong interfacial electric field inherently drives counteranions toward the electrode surface; however, single‐anion electrolytes tend to form disordered and thick interfacial layers because they lack the steric and adsorption‐energy diversity required for ordered packing [22]. Consequently, these additives cannot fully control the interfacial environment, resulting in insufficient hydrophobicity and weak directional regulation of Zn^2+^ flux [23, 24]. These shortcomings underscore the urgent need for interfacial strategies that achieve denser occupation of the IHP and maximize the functional roles of ions. This inspires the exploration of introducing multiple additives with varying molecular sizes and adsorption energies to enrich the IHP and create interfacial ion crowding, which could simultaneously optimize Zn^2+^ deposition orientation and water exclusion, an unprecedented capability that remains largely unexplored in the pursuit of reversible Zn metal deposition.

To address these limitations, we design a gradient‐boosted electrolyte, denoted as F‐ZnSO_4_, by introducing low‐concentration additives (5 mm each, ∼0.25% of the 2 M ZnSO_4_ baseline) of Zn(OTf)_2_, Zn(BF_4_)_2_, and Zn(TFSI)_2_. The coexistence of fluorine‐containing anions with different sizes creates a crowded, fluorine‐rich interfacial layer within the IHP. Simulations and experiments reveal that interfacial ion crowding leads to a densely packed and fluorine‐rich Helmholtz layer that enriches Zn^2+^ near the electrode and homogenizes the local ionic flux, thereby enabling stable and oriented Zn deposition. Electrochemical measurements further demonstrate long‐term stability, with Zn||Zn symmetrical cells stably operating for over 1,200 h at 5 mA cm^−2^ and 1,800 h at 10 mA cm^−2^, whereas cells with the baseline electrolyte fail in less than 100 h. These findings establish interfacial ion crowding as a fundamental design principle that offers a promising pathway toward durable and high‐performance AZMBs.

## 2 Results and Discussions

**FIGURE 1:**
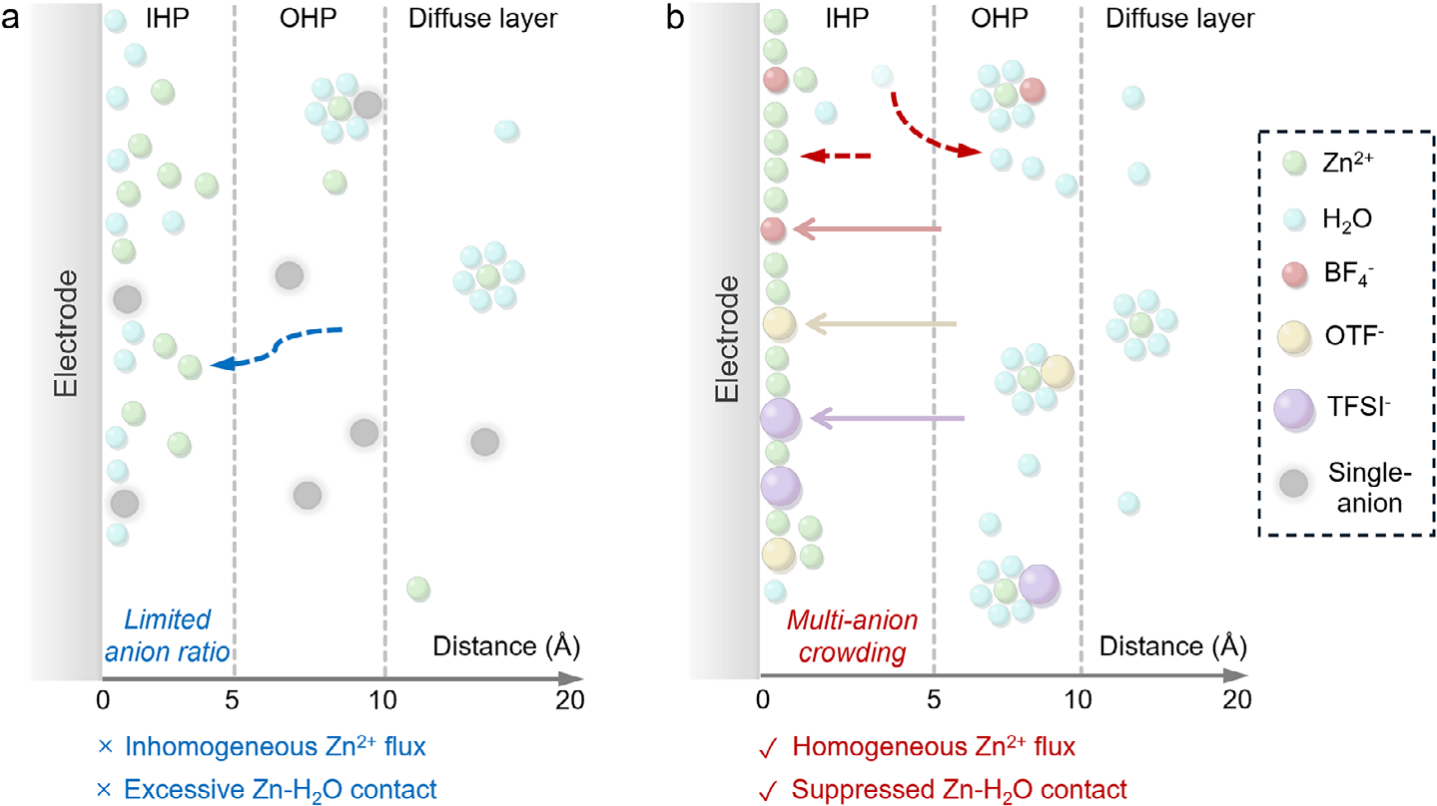
Schematic comparison of interfacial regulation in (a) single‐anion electrolytes and (b) fluorinated multi‐anion crowding at the IHP.

The interfacial behavior of different electrolyte configurations is schematically illustrated in Figure 1. In the single‐additive case (Figure 1a), Zn^2+^ ions accumulate near the negatively polarized Zn electrode, forming an IHP that remains largely occupied by water molecules. Hydrated Zn^2+^ and additional H_2_O molecules extend into the outer Helmholtz plane (OHP) [25, 26]. In this condition, the limited additive population within the double layer competes with water molecules for interfacial sites. Consequently, single‐additive systems provide insufficient hydrophobicity and weak regulation of Zn^2+^ flux, failing to mitigate parasitic HER or dendrite growth.

In contrast, the F‐ZnSO_4_ system (Figure 1b) introduces multiple fluorinated anions of different sizes and adsorption strengths, which collectively crowd the IHP and preferentially occupy interfacial sites. The enhanced effectiveness of the multicomponent anion system originates from the complementary interfacial roles of the three anions. In the mixed‐anion system: The TFSI^−^ anion provides a spatial framework at the interface due to its large size (0.326 nm); the small BF_4_^−^ anion (0.227 nm) fills the remaining free volume, increasing packing density; and the medium‐sized OTf^−^ anion (0.267 nm) modulates interfacial polarization and dipole orientation [27] (Figure S1).

TFSI^−^ is highly polarizable and forms a soft, weakly coordinating screening layer; BF_4_^−^ carries localized charge and interacts strongly with Zn^2+^; and OTf^−^ exhibits intermediate characteristics. When combined, these anions assemble into a layered double‐dipole interface, in which a highly polarizable outer layer and a charge‐dense inner layer cooperatively stabilize the IHP electric field and reduce the local electrostatic field experienced by interfacial water [28]. The combination of anions of different sizes enables more efficient spatial packing at the IHP, yielding a water‐excluding interfacial structure.

Such complementary electrostatic roles generate a balanced interfacial electric field and promote the formation of a layered dipole environment that attenuates the local electrostatic field experienced by interfacial water, thereby suppressing the HER, giving rise to more controlled Zn nucleation and deposition. In addition, the mixed‐anion configuration reshapes the coordination dynamics near the interface. Variations in anion size and binding strength inhibit the formation of the rigid, uniform coordination motifs characteristic of single‐anion systems, instead yielding an interfacial coordination network that is both highly covered and dynamically exchangeable. Simultaneously, the cooperative distribution of fluorinated anions minimizes aggregation and establishes a uniform Zn^2+^ flux toward the electrode.

**FIGURE 2:**
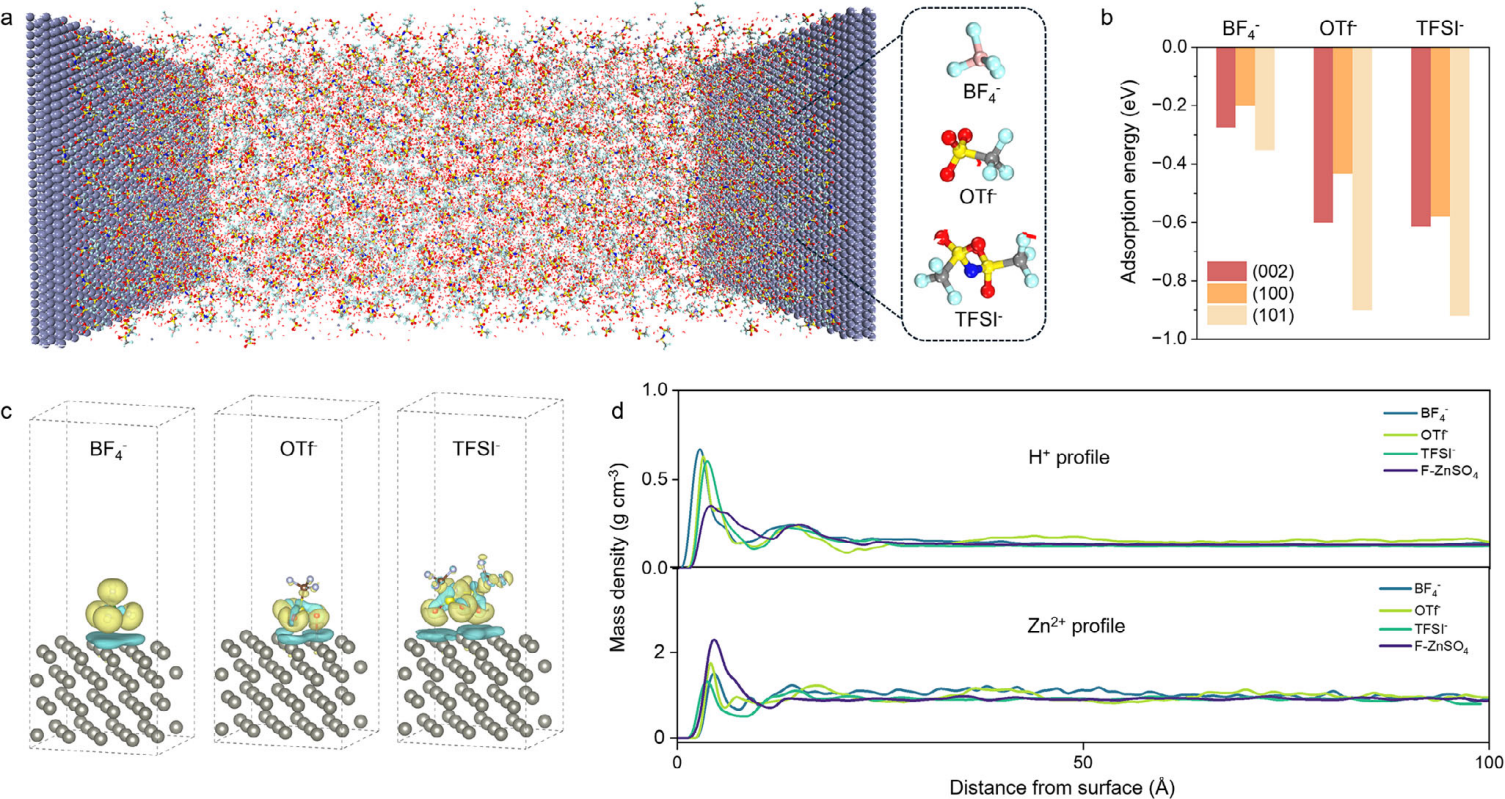
Theoretical calculation of electrolytes structures. (a) MD simulation snapshot of F‐ZnSO_4_ (2 M ZnSO_4_ with 5 mm each of Zn(OTf)_2_, Zn(BF_4_)_2_, and Zn(TFSI)_2_), with representative additive molecules shown in the dotted box. (b) Adsorption energies of BF_4_^−^, OTf^−^, and TFSI^−^ on different Zn crystal planes. (c) The charge density difference plots for BF_4_^−^, OTf^−^, and TFSI^−^ adsorbed on the (100) slab, where yellow and blue regions denote electron accumulation and depletion, respectively. (d) Mass density profiles of H^+^ (top) and Zn^2+^ (bottom) in the interfacial region as a function of distance from the Zn surface.

To gain mechanistic insight, molecular dynamics (MD) and density functional theory (DFT) calculations were performed. The mixed fluorinated‐anion electrolyte with a 1:1:1 ratio effectively suppresses anion aggregation and forms a more homogeneous fluorinated layer at the Zn surface (Figure 2a). The adsorption energies of different anions on Zn crystal planes were then calculated (Figures 2b and S2), showing an increasing order of BF_4_^−^ < OTf^−^ < TFSI^−^. Such a gradient may facilitate more complete interfacial coverage, thereby homogenizing Zn^2+^ nucleation sites. Among the Zn facets, the (100) surface exhibits the weakest Zn^2+^ adsorption energy (stronger affinity) than the (002) and (101) surfaces, thus avoiding either unstable or irreversible binding, and favoring reversible Zn deposition along the (100) orientation, a direction associated with suppressed dendrite growth. Extended computational analyses of ion adsorption, surface‐binding configurations, bulk solvation characteristics, and interfacial electrostatic profiles are provided in Supplementary Note 1.

To further elucidate interfacial interactions, differential charge density plots were analyzed (Figure 2c). The extent of charge transfer between adsorbed anions and the Zn (100) surface follows the same order (BF_4_^−^ < OTf^−^ < TFSI^−^), indicating progressively stronger surface bonding and stable anchoring of these anions.

The fluorinated system used in this work serves as a practical model for revealing this mechanism, owing to its well‐defined interfacial affinity and strong ability to reorganize the near‐surface ion environment. Importantly, the underlying interfacial ion crowding principle is not unique to fluorinated species and can, in principle, be extended to other anion systems.

The zeta potential measurement provides an additional macroscopic perspective on interfacial stability. Compared with ZnSO_4_ (4.6 mV), F‐ZnSO_4_ exhibits a markedly higher value of 19.9 mV (Table S1). A higher positive zeta potential, although reflecting the potential at the slipping plane rather than directly at the IHP, generally indicates a more positively charged slipping plane and hence a more electrostatically stabilized double layer. This interpretation is consistent with the MD and DFT results (Figures 2a–c and S2), which reveal cooperative and orderly anion adsorption that prevents excessive charge neutralization and facilitates a more homogeneous Zn^2+^ flux toward the surface [29, 30]. The decrease in EDL capacitance (Figure S3) is consistent with the lower adsorption energies obtained from MD/DFT calculations, as stronger (lower energy) anion adsorption forms a denser and less polarizable Helmholtz layer. This trend is further supported by the intermediate capacitance of the single‐anion electrolyte (Figures S4 and S5), confirming that multi‐anion cooperation can generate the more tightly packed Helmholtz layer observed in the mixed‐anion system.

To evaluate the practical consequence of this species arrangement, we simulated the mass density profiles of H^+^ and Zn^2+^ across the interface (Figure 2d). In F‐ZnSO_4_, H^+^ density near the surface is substantially suppressed compared to single‐anion electrolytes, suggesting reduced proton accessibility to the IHP, while Zn^2+^ density is significantly enhanced, favoring rapid nucleation and reversible deposition. Collectively, these results demonstrate that the cooperative adsorption of fluorinated anions produces a stable gradient interfacial layer that both hinders water access and promotes homogeneous Zn^2+^ enrichment near the surface.

In contrast to bulk‐electrolyte modification strategies, the results further suggest that the fluorinated system does not induce a substantial change in the solvation structure of Zn^2+^. Consistently, ^1^H NMR shows only slight proton chemical‐shift changes among ZnSO_4_, F‐ZnSO_4_, and the single‐additive electrolytes (Figure S6), while ^19^F NMR provides complementary evidence for the fluorinated‐anion environment in F‐ZnSO_4_ (Figure S7). In F‐ZnSO_4_, the blue‐shifted O‐H stretching bands observed in Fourier transform infrared spectroscopy (FTIR) (Figure S8) together with Raman spectra exhibit only minimal chemical‐shift variations (Figure S9), consistent with the negligible changes observed in ionic conductivity (Figure S10 and Table S2) and viscosity (Figure S11) [31], show negligible perturbation to bulk O─H vibrations, indicating that the solvent environment in the electrolyte remains essentially unchanged [32, 33, 34, 35]. These observations collectively demonstrate that the performance enhancement originates from interfacial regulation rather than bulk solvation changes. Therefore, the anions anchored in the IHP dominate interfacial stabilization, restructure interfacial charge distribution, suppress water accessibility, and the diffuse layer also contributes to promoting Zn^2+^ deposition.

### 2.1 Electrochemical Performance

**FIGURE 3:**
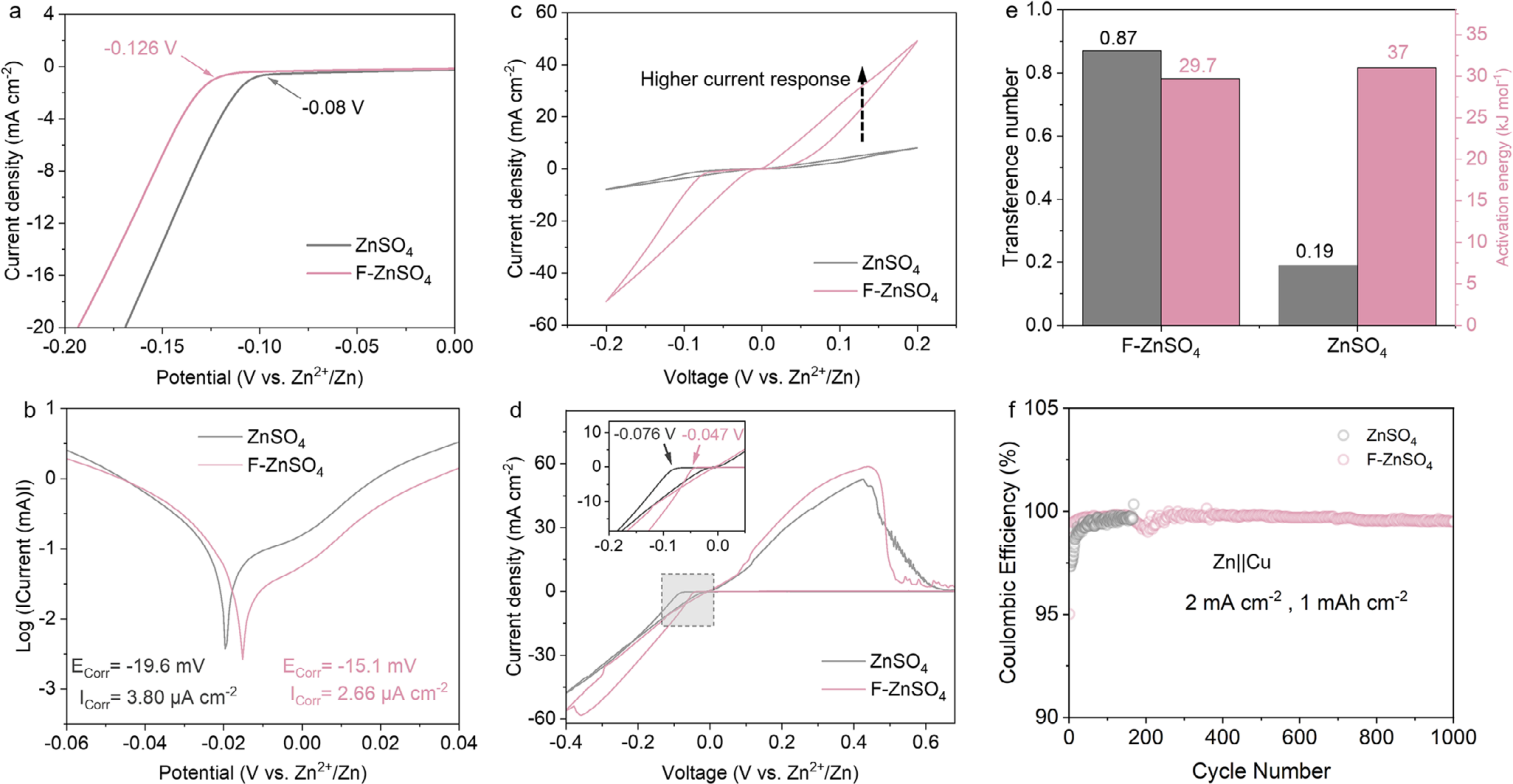
Electrochemical investigation of F‐ZnSO_4_ and ZnSO_4_ electrolytes. (a) LSV curves at a scan rate of 5 mV s^−1^. (b) Linear polarization curves at a scan rate of 5 mV s^−1^. (c) Cyclic voltammetry (CV) curves of Zn||Zn symmetric cells at a scan rate of 0.2 mV s^−1^. (d) CV curves of Zn||Ti asymmetric cells at a scan rate of 0.2 mV s^−1^. (e) Zn^2+^ transference number and interfacial activation energies (see Figures S13–S16 for details). (f) Coulombic efficiency (CE) of Zn||Cu coin cells at 2 mA cm^−2^ with a fixed capacity of 1 mAh cm^−2^.

The electrochemical stability of F‐ZnSO_4_ and ZnSO_4_ electrolytes was first compared using linear sweep voltammetry (LSV, Figure 3a). The onset potential for HER in F‐ZnSO_4_ is significantly more negative (−0.126 V) than in ZnSO_4_ (−0.08 V), demonstrating that the fluorinated electrolyte effectively suppresses HER at the Zn surface [36, 37]. To further decouple the effect of individual anions, HER tests were also carried out in electrolytes containing a single fluorinated additive (Figure S12). The onset potentials in BF_4_^−^, OTf^−^, and TFSI^−^ electrolytes are −0.104 V, −0.1 V, and −0.106 V, respectively, all less negative than that of the mixed F‐ZnSO_4_ system. This indicates that while single anions can partially expel water molecules from the IHP, their effect is limited. Only the cooperative adsorption of multiple fluorinated anions produces a robust hydrophobic interface that efficiently blocks water access and maximally suppresses HER, consistent with the simulation results (Figure 2d).

Linear polarization curves further reveal that the corrosion potential of Zn in F‐ZnSO_4_ shifts positively (−15.1 mV vs. −19.6 mV), while the corrosion current density decreases from 3.80 µA cm^−2^ in ZnSO_4_ to 2.66 µA cm^−2^ in F‐ZnSO_4_ (Figure 3b). These results indicate that the fluorine‐rich IHP effectively protects the Zn surface against parasitic corrosion.

Symmetric and asymmetric cell tests further demonstrate the kinetic benefits of the modified IHP. From the cyclic voltammetry (CV) curves of Zn||Zn cells, F‐ZnSO_4_ delivers stronger and more symmetric redox responses (Figure 3c). Moreover, the nucleation potential of Zn on a Ti substrate is markedly reduced (−0.047 V in F‐ZnSO_4_ vs. −0.076 V in ZnSO_4_, Figure 3d), reflecting facilitated Zn^2+^ flux and a lowered nucleation barrier at the modified IHP.

The Zn^2+^ transport properties were further examined under constant polarization. Electrochemical impedance spectroscopy (EIS) spectra and *I*‐*t* curves before and after polarization are shown in Figures S13 and S14. The Zn^2+^ transference number was calculated using the formula [38]:

(1) $$t_{zn^{2+}}=\frac{I_{S}\left(\Delta V-R_{0}I_{0}\right)}{I_{0}\left(\Delta V-R_{S}I_{S}\right)}$$

Where ΔV denotes the polarization potential of 10 mV, I_0_ and I_S_ represent the original and steady‐state currents, respectively, and R_0_ and R_S_ denote the charge transfer resistance (R_ct_) measured by EIS. The t_Zn_^2+^ values for F‐ZnSO_4_ were calculated to be as high as 0.87 (Figure S13), while the corresponding value for ZnSO_4_ was 0.19 (Figure S14). The significantly increased apparent t_Zn_^2^^+^ value of F‐ZnSO_4_ suggests altered ion‐transport behavior and an enhanced contribution of Zn^2^^+^ to charge transport under the tested conditions, which is consistent with the interfacial regulation induced by the fluorinated IHP. It should be noted that the transference number obtained from the Bruce–Vincent–Evans method in aqueous Zn systems is used here primarily for relative comparison under identical testing conditions rather than as an absolute descriptor of Zn^2^^+^ transport. Temperature‐dependent EIS combined with Arrhenius equation [39, 40] (Figures S15 and S16) further revealed a lower interfacial activation energy (29.7 kJ mol^−1^ vs. 37.0 kJ mol^−1^), indicating reduced charge transfer barrier and a more favorable ion migration process.

Moreover, Zn||Cu cells demonstrated the practical advantages of the fluorinated interface. In F‐ZnSO_4_, Coulombic efficiency (CE) was stably maintained at 99.7% for over 1000 cycles (Figure 3f), in sharp contrast to ZnSO_4_, which failed within 200 cycles due to severe side reactions and irreversible Zn anode degradation. The superior performance of F‐ ZnSO_4_ can thus be attributed to the crowding fluorinated IHP, which synergistically regulates Zn^2+^ flux, suppresses parasitic reactions, and enhances both cycling durability and reversibility.

**FIGURE 4:**
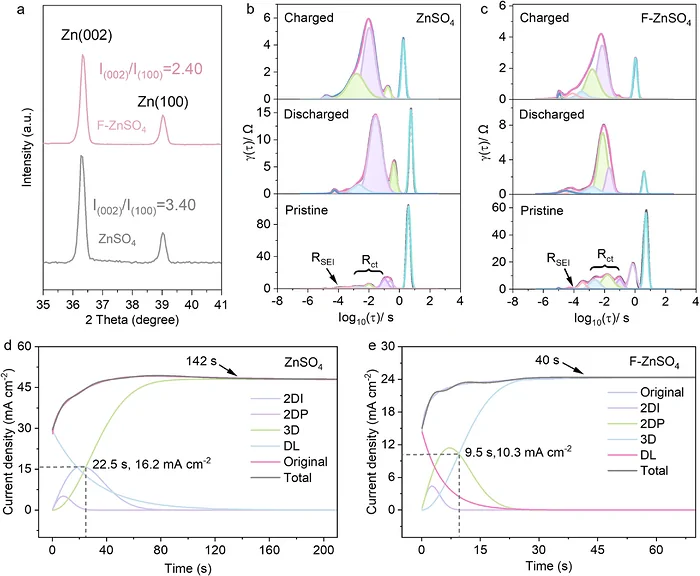
Zn deposition behavior in F‐ZnSO_4_ and ZnSO_4_ electrolytes. (a) GI‐XRD patterns of Zn foils after 20 plating/stripping cycles in ZnSO_4_ and F‐ZnSO_4_ electrolytes (b, c). The distribution of relaxation times (DRT) plot of the in situ electrochemical impedance spectroscopy (EIS), attributed to (b) ZnSO_4_ and (c) F‐ZnSO_4_. (d, e) Chronoamperogram (CA) attributed to (d) ZnSO_4_ and (e) F‐ZnSO_4_ at an overpotential of −150 mV.

Grazing incidence X‐ray diffraction (GIXRD) was employed to analyze the surface texture of Zn deposits. As shown in Figure 4a, after plating and stripping at a current density of 2 mA cm^−2^, the intensity ratio of Zn (002) to Zn (100) in F‐ZnSO_4_ electrolyte reaches 2.4, compared to 3.4 in the ZnSO_4_ electrolyte. This enhancement of the (100) plane in the fluorinated system suggests that F‐ion‐induced gradients suppress local aggregation and allow for more homogeneous electrolyte dispersion, thereby promoting vertical Zn growth [41, 42, 43, 44], consistent with the growth orientation schematic (Figure S17). After 100 cycles, the GIXRD (Figure S18) shows a further decrease of the (002)/(100) ratio to 2.14, reaffirming the strengthened (100)‐preferred growth in F‐ZnSO_4_. The corresponding SEM image (Figure S19) shows a smoother and more vertically aligned surface texture, in line with the enhancement of the (100) facet contribution.

To further elucidate interfacial behavior, in situ EIS data were analyzed by the distributed relaxation time (DRT) method, which decouples electrochemical processes by characteristic relaxation times [45, 46]. As shown in Figure 4b,c, the F‐ZnSO_4_ electrolyte exhibits lower and more stable charge transfer resistance across pristine, discharged, and charged states, implying suppressed by‐product formation and smoother Zn^2+^ migration/nucleation. Additional peaks in the pristine‐state spectrum (Figure 4c) arise from the interaction of diverse F‐containing anions. After 10 times cycling, strong C 1s and F 1s signals (Figures S20 and S21) confirm the incorporation of F‐containing interfacial species. Additional ex situ XPS and time‐dependent Raman analyses in Supplementary Note 2 further support the formation of a stable fluorinated interphase during Zn deposition.

To probe nucleation dynamics, chronoamperometry was analyzed via nonlinear least squares (NLLS) fitting [47, 48]. As shown in Figure 4d,e, Zn deposition in ZnSO_4_ reached the 3D diffusion regime after 22.5 s, whereas F‐ZnSO_4_ transitioned within only 9.5 s under the same overpotential (−150 mV). The shortened two‐dimensional (2D) nucleation period in F‐ZnSO_4_ suggests a reduced nucleation barrier, limiting dendritic grain aggregation.

Moreover, the smaller nucleation current observed in F‐ZnSO_4_ suppresses uneven Zn growth, since inhomogeneous 2D nucleation generally leads to loose and disordered deposits. Once the steady‐state stage is reached, a dynamic balance among nucleation, growth, and ion transport is established. The steady‐state current in F‐ZnSO_4_ is lower than in ZnSO_4_, attributable to the shortened 2D nucleation stage, which reduces the number of nucleation sites and consequently decreases dendrite formation.

**FIGURE 5:**
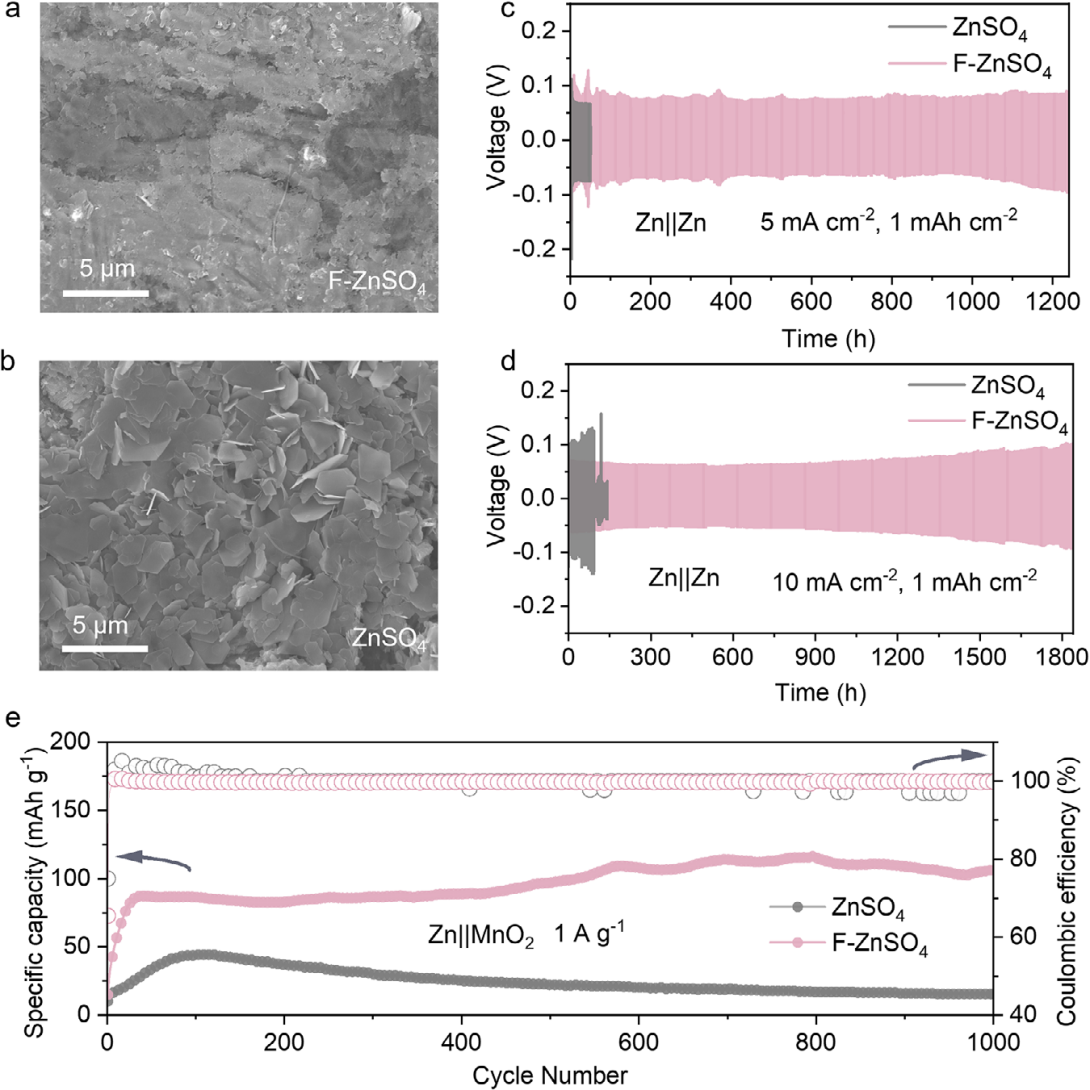
Post‐mortem morphology and cycling performances with ZnSO_4_ and F‐ZnSO_4_ electrolytes. (a, b) SEM images of the Zn foils cycled with (a) F‐ZnSO_4_ and (b) ZnSO_4_ electrolytes after 20 cycles at 2 mA cm^−2^. (c, d) Galvanostatic cycling curves of Zn||Zn symmetric cells at (c) 5 mA cm^−2^ and (d) 10 mA cm^−2^ with a fixed capacity of 1 mAh cm^−2^. (e) Long‐term cycling properties of Zn||MnO_2_ full cells at 1 A g^−1^.

The morphological evolution was tracked by in situ optical microscopy (Figure S22). After 30 min plating at 10 mA cm^−2^, deposits in the ZnSO_4_ electrolyte were almost entirely covered by dendrites, whereas those in the F‐ZnSO_4_ produced uniform, compact deposits. Post‐mortem SEM imaging (Figures 5a,b and S23) reveals smoother Zn foil surfaces after 20 cycles in F‐ZnSO_4_, confirming mitigated formation of zinc hydroxide sulfate (ZHS) associated with HER and a reduced risk of short‐circuiting.

The F‐ZnSO_4_ electrolyte used in this study is anticipated to provide superior electrochemical performances due to its distinguished properties. As shown in Figure S24, Zn||Zn symmetric cells cycled at 2 mA cm^−2^ with a capacity of 1 mAh cm^−2^ maintained stable operation for 1050 h with an overpotential of around 50 mV. In contrast, cells with ZnSO_4_ electrolytes failed within 440 h, possibly due to the dendrite‐induced short circuits. Under a higher current density of 5 mA cm^−2^ (Figure 5c), the F‐ZnSO_4_ system achieved a lifespan of 1250 h, while ZnSO_4_ lasted less than 100 h. Even at 10 mA cm^−2^ (Figure 5d), Zn plating remained stable for over 1800 h. In addition, the F‐ZnSO_4_ electrolyte demonstrated excellent rate capability across a wide range of current densities (1–10 mA cm^−2^, 1 mAh cm^−2^), sustaining stable operation for up to 1100 h (Figure S25). These results suggest that F‐ZnSO_4_ effectively regulates Zn^2+^ flux, enabling highly durable and efficient Zn metal anodes.

To demonstrate practical applicability, Zn||MnO_2_ full cells were assembled with commercial MnO_2_ cathodes on carbon paper. From the CV curves, with increasing sweep rate, the ZHS‐related peak (Peak 1) diminishes, and the Mn^3+^/Mn^2+^ conversion peak (Peak 2) weakens (Figure S26), reflecting suppressed H^+^ involvement [49, 50, 51]. Since the protons in Zn(H_2_O)_6_ are highly labile [24, 52], their suppression in F‐ZnSO_4_ enhances reversibility. Consequently, the full cells retained ∼105 mAh g^−1^ after 1000 cycles at 1 A g^−1^ (Figure 5e), even without the addition of MnSO_4_, indicating the excellent stability of F‐ZnSO_4_. Stable cycling is also achieved in F‐ZnSO_4_‐based Zn||MnO_2_ full cells under deep charge/discharge at 0.2 A g^−1^ (Figure S27). Additional post‐mortem XRD analyses of both the Zn anode and MnO_2_ cathode after cycling are provided in Supplementary Note 3.

## 3 Conclusion

This work demonstrates that introducing multiple low‐concentration fluorinated anions into ZnSO_4_ electrolytes establishes a crowded, fluorine‐rich interfacial layer that cannot be achieved by single‐additive strategies. Cooperative adsorption within the inner Helmholtz plane establishes a densely packed interfacial layer that regulates Zn^2+^ coordination and directs preferential (100) deposition, leading to compact and dendrite‐free zinc growth. Supported by DFT, MD, and spectroscopic analyses, this interfacial ion crowding strategy suppresses parasitic reactions, elevates the Zn^2+^ transference number to 0.87 (vs. 0.19 for ZnSO_4_), and prolongs Zn||Zn cycling stability to over 1800 h, more than ten times that of the baseline electrolyte. In practical Zn||MnO_2_ full cells, it delivers highly reversible cycling for over 1000 cycles without Mn^2+^ additives, in sharp contrast to rapid failure in conventional ZnSO_4_. By demonstrating durable Zn deposition and stable full‐cell operation, this study positions interfacial fluorinated‐ion crowding as a cornerstone for the future design of robust aqueous zinc metal batteries.

## Author Contributions

**Xinyu Zhang**: methodology, formal analysis, investigation, writing – original draft. **Jitao Shang**: methodology and software. **Ruwei Chen**: resources and data curation. **Jianrui Feng**: resources and software. **Hang Yang**: resources and visualization. **Jingyi Wang**: resources and validation. **Fei Guo**: resources. **Shuhui Li**: resources. **Zijuan Du**: resources and validation. **Peie Jiang**: resources and formal analysis. **Xiaoxia Guo**: resources and formal analysis. **Wei Zhang**: resources and formal analysis. **Jie Chen**: resources and data curation. **Hongzhen He**: resources and data curation. **Xuan Gao**: resources, validation, and visualization. **Zhenjing Jiang**: resources, formal analysis, and software. **Bing Wang**: resources, investigation, and methodology. **Yuhang Dai**: resources, writing – review and editing, data curation, formal analysis, and supervision. **Guanjie He**: resources, funding acquisition, methodology, writing – review and editing, supervision, and project administration.

## Conflicts of Interest

The authors declare no conflicts of interest.

## Supporting information




**Supporting File**: anie73589‐sup‐0001‐SuppMat.docx.

## Data Availability

The data that support the findings of this study are available from the corresponding author upon reasonable request.
