# Supplementary material for: Interfacial Fluorinated Ion Crowding Enables Reversible Zinc Metal Batteries
Source: Angew Chem Int Ed Engl. 2026 Jul 20;65(39):e3251724. doi: 10.1002/anie.3251724 (PMC13592298; doi:10.1002/anie.3251724)
Supplement: Supplementary file 1 [file ANIE-65-e3251724-s001.docx]

**Supporting Information**

**Interfacial fluorinated ion crowding enables reversible zinc metal batteries**

Xinyu Zhang^1#^, Jitao Shang^2#^, Ruwei Chen^1^, Jianrui Feng^1^, Hang Yang^1^, Jingyi Wang^1^, Fei Guo^1^, Shuhui Li^1^, Zijuan Du^3^, Peie Jiang^4^, Xiaoxia Guo^5^, Wei Zhang^1^, Jie Chen^1^, Hongzhen He^1^, Xuan Gao^1,4^, Zhenjing Jiang^6^, Bing Wang^7^, Yuhang Dai^4,^*, Guanjie He^1,^*

1 Christopher Ingold Laboratory, Department of Chemistry, University College London, London WC1H 0AJ, UK

2 Institute of Technological Sciences, Wuhan University, Wuhan 430072, China

3 State Key Laboratory of Silicate Materials for Architectures, Wuhan University of Technology, Wuhan 430070, China

4 Department of Engineering Science, University of Oxford, Oxford OX1 3PJ, UK

5 The Electrochemical Innovation Lab, Department of Chemical Engineering, University College London, London WC1E 6BT, United Kingdom

6 SEU-FEI Nano-Pico Center, Key Laboratory of MEMS of Ministry of Education, Southeast University, Nanjing 210096, China

7 State Key Laboratory of Advanced Technology for Materials Synthesis and Processing, Wuhan University of Technology, Wuhan 430070, China

^#^ These authors contributed equally to this work

^*^Email: dai.yuhang.72z@st.kyoto-u.ac.jp; [g.he@ucl.ac.uk](mailto:g.he@ucl.ac.uk).

**Experimental section**

***Electrode and electrolyte preparation***

The baseline electrolytes (BE, 2M ZnSO_4_) were obtained by dissolving 0.5 mol ZnSO_4_·7H_2_O (>99%, VWR chemicals) into 250 mL deionized water under constantly magnetic stirring for 30 min. The designed electrolyte (F-ZnSO_4_) was formulated by adding 5 mM Zn(CF_3_SO_3_)_2_ (i.e., Zn(OTf)_2_), 5 mM Zn(BF_4_)_2_·*n*H_2_O, and 5 mM zinc di[bis(trifluoromethylsulfonyl)imide] (i.e., Zn(TFSI)_2_, 95%, Sigma-Aldrich) into the 2 M ZnSO_4_ solution.

Zinc electrodes were obtained by polishing zinc foil (99.99%, 70 um) with 1000-grit sandpaper. Copper electrodes were cut from Cu foils (99.99%). MnO_2_ electrodes were fabricated by 70 wt.% commercial MnO_2_ Powder (Sigma-Aldrich), 20 wt.% active carbon (YP-80F, Kuraray Chemical) and 10 wt.% PVDF (Sigma-Aldrich) in N-methyl-2-pyrrolidine (NMP, ≥ 99%, Sigma-Aldrich). The slurry was cast on hydrophilic carbon cloth (Toray) and dried in vacuum oven for 8 h. The mass loading of MnO_2_ was controlled at 0.9-1.1 mg cm^-2^.

***Materials characterizations***

Scanning electron microscopy (SEM, JEOL-JSM-7600F, 15.0 kV) was used to investigate the morphologies of samples. In situ optical microscopy was conducted by a VisiScope® BL254 T1 (VWR) instrument in Zn||Zn symmetric cells with different electrolytes. Grazing-incidence X-ray diffraction (GIXRD) was performed on a PANalytical Empyrean device (Cu Kα radiation; 40 kV, 40 mA) over 5~80° (step size: 0.05 °, scan rate: 0.1° s^-1^). Raman spectra were recorded on a LabRAM HR Evolution instrument. (laser wavelength: 532 nm). FTIR was conducted on a BRUKER ATR-FTIR spectrometer (platinum-ATR). X-ray photoelectron spectroscopy (XPS) was performed on a Thermo Scientific K-Alpha photoelectron spectrometer. The Zeta potentials were collected using Zetasizer version 7.13.

***Electrochemical characterizations***

The Zn||Zn symmetric cells were assembled in CR2032 coin cells using zinc foil (70 μm thick, 16 mm diameter) as both electrodes, a glass fiber separator (GF/A, Whatman, 19 mm diameter), and 100 μL of electrolyte. Zn||Cu asymmetric cells were prepared with zinc foil (70 μm thick, 16 mm diameter) as the anode, copper foil (12 mm diameter) as the cathode, the same glass fiber separator, and 100 μL of electrolyte. Zn||MnO_2_ full cells were assembled with zinc foils (70 μm thick, 16 mm diameter) as the anode, MnO_2_ cathodes coated on carbon paper (10 mm), a glass fiber separator (19 mm), and 100 μL of electrolyte. The cycling performance of the cells were conducted on a Neware battery test system (Shenzhen, China).

The electrochemical impedance spectroscopy (EIS) tests, chronoamperogram (CA), linear polarization curves (LSV) were achieved by a VMP3 Biologic workstation. The activation energy was obtained by EIS test. By testing the impedance of the coins at different temperatures ranging from 288 to 328 K, the activation energy can be obtained by the equation below:

The Arrhenius equation: −𝑙𝑛𝑅𝑐𝑡 = 𝑙𝑛𝐴 − 𝐸𝑎/𝑅T

In this equation: Ea represents the activation energy. A represents the frequency factor, R represents the gas constant, *T* represents the absolute temperature.

The in-situ EIS test was set to first discharge for 1 h and then charge for 1 h at a current density of 1 mA cm^-2^. The CA test used Zn||Ti symmetric cells at an overpotential of -150 mV, and LSV curves based on Zn||Ti half cells at 5 mV s^-1^.

Electrolyte viscosity was measured using KINEXUS rotational rheometer equipment. Measurements were conducted at 25 °C with a constant shear rate of 20 s^-1^ to ensure stable flow conditions and reproducible viscosity readings.

Ionic conductivity was measured using a Swagelok‐type Cell. Whatman GF-D separator (12 mm diameter) was used as the ion‐transport membrane. The resistance (Rₛ) was obtained from the high frequency intercept of the Nyquist plot, and the ionic conductivity was calculated using *σ* =*d*/ (*R*×*A*), where d is the electrode spacing and *A* is the geometric area determined by the 12 mm separator diameter.

The capacitance (C) was obtained from the linear relationship between the capacitive current density (*j*₍c₎) and the scan rate (v). The capacitive current density was extracted as $j_{c}=(j_{+}-j_{-})/2$, where $j_{+}$and $j_{-}$are the current densities during the forward and reverse scans, respectively. The areal capacitance was calculated using:

$$C^{'}=\frac{j_{c}}{v}$$

CV scans were performed at scan rates of 1, 3, 5, 10 and 20 mV s^-1^ between -20 and 20 mV using Zn||Zn symmetric cells.

***Computational methods and models***

All molecular dynamics (MD) simulations were performed using the LAMMPS package.^[1]^ To ensure a physically meaningful description of the electrochemical boundary condition, the interfacial system was simulated under a constant-potential framework using the CONP module, where the electrode atomic charges are dynamically adjusted at each MD step to satisfy the prescribed electrostatic potential of the metallic electrodes.^[2-5]^ This approach enables a self-consistent treatment of electrode polarization and avoids the limitations of fixed-charge or vacuum-referenced potential definitions.

**Model construction and simulation cell**

The simulated electrochemical cell consists of two parallel metallic electrodes separated by an aqueous electrolyte region. The electrodes were modeled as two rigid Zn slabs positioned at the bottom and top of the simulation box along the surface-normal direction (defined as the *z*-axis). The electrolyte region between the electrodes contains explicit water molecules and dissolved ionic species, including Zn^2+^ cations and the corresponding counterions. Periodic boundary conditions were applied in the lateral directions (*x* and *y*), while the system remained periodic along *z* to maintain compatibility with the long-range electrostatics implementation and the constant-potential algorithm. To mimic metallic electrodes, two Zn slabs were treated as electrode atoms within the CONP model. The electrode atoms were assigned to two separate groups (bottom and top electrodes). During production runs, the Zn slabs were kept rigid (harmonically restrained) to represent a mechanically stable electrode substrate while allowing the electrolyte molecules and ions to evolve freely. Specifically, the Zn electrode atoms were either fully frozen by setting their velocities to zero and excluding them from time integration or constrained with strong positional restraints to suppress drift while maintaining realistic surface corrugation. Constant-potential simulations were conducted using the CONP method implemented in LAMMPS, in which the instantaneous electrode atomic charges $\left\{ q_{i} \right\}$are optimized under the constraint that the anode remains at a fixed electric potential of -0.05V.

**Force field description**

Non-bonded interactions were represented using a combination of Lennard–Jones (LJ) and Coulombic terms. LJ interactions were described by the 12–6 potential:

$$U_{\mathrm{LJ}}(r)=4\varepsilon\left[ \left( \frac{\sigma}{r} \right)^{12} - \left( \frac{\sigma}{r} \right)^{6} \right]$$

Long-range Coulomb interactions were computed using PPPM/electrode with the same cutoff as the LJ interactions.^[6]^ Water molecules were modeled using a rigid TIP3P-type model,^[7]^ with intramolecular O–H bonds and H-O-H angle constrained using SHAKE.^[8]^ The force field of the ionic species was represented with common classical electrolyte force fields.^[9-12]^ All simulations were carried out in the real unit system. The integration time step was set to 0.5fs. After initial energy minimization, the systems were equilibrated under canonical (NVT) conditions. Temperature was maintained at 300 K using a Nosé–Hoover thermostat applied only to the electrolyte atoms.^[13]^ Equilibration was performed for 1000 ps, followed by production simulations of 5ns. Trajectories were saved every 2000 steps for analysis. The stability of the constant-potential simulation was confirmed by monitoring the instantaneous electrode potentials reported by CONP, the total electrode charges, and the convergence of electrolyte structural observables.

The planar-averaged electrostatic potential $\phi(z)$and electric field $E(z)$were obtained to directly address the spatial distribution of the potential drop in the electrochemical cell. The charge density profile $\rho(z)$was computed from the bin-averaged ionic charge distribution:

$$\rho(z)=\sum_{i} q_{i}\text{ }\langle n_{i}(z)\rangle,$$

where $q_{i}$and $n_{i}(z)$denote the charge and number density of species $i$, respectively. The electrostatic potential profile was then obtained by solving the one-dimensional Poisson equation under appropriate boundary conditions consistent with metallic electrodes:

$$\frac{d^{2}\phi(z)}{dz^{2}}=-\frac{\rho(z)}{\varepsilon_{0}},$$

and the electric field was calculated as:

$$E(z)=-\frac{d\phi(z)}{dz}.$$

Importantly, the theoretical potential was referenced to the bulk electrolyte plateau region, where $\phi(z)$is spatially converged and $d\phi/dz\approx0$. In practice, $\phi_{\mathrm{bulk}}$was defined as the average potential within a central window of the electrolyte (e.g., $z\in[z_{1},z_{2}]$, far from both electrodes), and the relative potential profile was reported as $\Delta\phi(z)=\phi(z)-\phi_{\mathrm{bulk}}$. This bulk-referenced definition enables a direct comparison with experimentally defined electrode potentials and avoids the unphysical assumption of a uniform potential gradient across the entire electrode separation.

Detail of the distribution of relaxation times (DRT) analysis of EIS spectra Drt analysis is achieved by DRTtools (https://github.com/ciuccislab/DRTtools) which supported by Dr.Francesco Ciucci. Gaussian discretization and hierarchical Bayesian methods were used for analytical runs on EIS data.

The detailed fitting process used in CA analysis: The chronoamperometry (CA) transients were analyzed using a nucleation-growth kinetic model that decomposes the measured current into four physically meaningful contributions:

$I=I_{DL}+I_{2DI}+I_{2DP}+I_{3DP}$	(1)

These contributions are defined explicitly as:

$I_{DL}=k_{D1}exp\left( -k_{D2}t \right)$	(2)

$I_{2DI}=\frac{2\pi nFMhNk^{2}t^{2}}{\rho}exp\left( -\frac{\pi NM^{2}k^{2}t^{2}}{\rho^{2}} \right)$ (3)

$I_{2DP}=\frac{\pi nFMhANk^{2}t^{2}}{\rho}exp\left( -\frac{\pi NM^{2}Ak^{2}t^{3}}{{3\rho}^{2}} \right)$ (4)

$I_{3DP}=nFk\left[ 1-exp\left( -\frac{\pi NM^{2}Ak^{2}t^{3}}{{3\rho}^{2}} \right) \right]$ (5)

The parameters used in the above equations are defined as follows: the number density of active sites (*N*), the growth rate constant (*k*), the nucleation rate constant (*A*), the molecular weight (*M*), density (*ρ*), deposited layer thickness (*h*), and the molar charge transferred (*nF*). Most of the parameters are unknown or difficult to measure, so the following equations are used for fitting with only eight independent parameters:

$I=p_{1}exp\left( -p_{2}t \right)+p_{3}t^{2}exp\left( p_{4}t^{2} \right)+p_{5}t^{2}exp\left( p_{6}t^{3} \right)+p_{7}\left[ 1-exp\left( {p_{8}t}^{3} \right) \right]$ (6)

**Figure S1.** Molecular structures of the three zinc salts used in this study: Zn(TFSI)_2_, Zn(OTf)_2_, and Zn(BF_4_)_2_.

**Figure S2.** The comparison of the adsorption energy when different ions were adsorbed on different surfaces of the Zn by force field and DFT, individually.

**Figure S3.** Cyclic voltammograms of symmetric Zn||Zn cells in (a) ZnSO_4_ and (b) F-ZnSO_4_ electrolytes recorded at various scan rates in a narrow voltage range of -20 to 20 mV. (c) EDL capacitance obtained by plotting the capacitive current versus scan rate for both electrolytes.

**Figure S4.** Cyclic voltammograms of symmetric Zn||Zn cells in (a) ZnSO_4_ +5mm Zn(OTF)_2_ electrolytes recorded at various scan rates in a narrow voltage range of -20 to 20 mV.(b) EDL capacitance obtained by plotting the capacitive current versus scan rate.

To further isolate the role of cooperative adsorption, we additionally measured the capacitance of an electrolyte containing only a single fluorinated additive (2M ZnSO_4_ + 5 mM Zn(OTf)_2_). Its intermediate capacitance (35.09 μF cm^-2^) falls between ZnSO_4_ and F-ZnSO_4_, indicating that while single-anion adsorption contributes to interfacial structuring, it remains less effective than the cooperative packing achieved in the mixed-anion system. Demonstrating that multi-anion cooperation produces a more tightly packed Helmholtz layer than any single-additive formulation.

**Figure S5.** Comparison of the EDL capacitance (C) for the three electrolytes.

**Figure S6.** ¹H NMR chemical shifts of ZnSO_4_, F-ZnSO_4_ electrolyte, and electrolytes with individual additives of Zn(OTf)_2_, Zn(BF_4_)_2_, and Zn(TFSI)_2_, respectively.

**Figure S7.** ^19^F NMR spectra for F-ZnSO_4_ electrolyte.

**Figure S8.** FT-IR spectra for ZnSO_4_ and F-ZnSO_4_ electrolytes.

**Figure S9.** Raman spectra of ZnSO_4_ and F-ZnSO_4_ electrolytes.

**Figure S10.** Electrochemical impedance spectra (EIS) of the ZnSO_4_ electrolyte and F-ZnSO_4_ electrolyte. The solution resistance (R_S_), determined from the high-frequency interception of the Nyquist plots, was used to calculate the ionic conductivity.

**Figure S11.** Viscosity of the ZnSO_4_ and F-ZnSO_4_ electrolytes

**Figure S12.** LSV curves showing HER in F-ZnSO_4_ electrolyte and in electrolytes with single additives of Zn(OTf)_2_, Zn(BF_4_)_2_, and Zn(TFSI)_2_.

**Figure S13.** Impedance spectra of F-ZnSO_4_ before and after polarization (purple and blue, respectively). The inset shows the current-time response during polarization at 10 mV.

The transference number was determined using the Bruce–Vincent method, where the current values were extracted at two well-defined regions of the CA trace: point A, corresponding to the onset of the quasi-steady decay following the initial transient, and point B, representing the fully established steady-state plateau.

**Figure S14.** Impedance spectra of ZnSO_4_ before and after polarization (gray and dark, respectively). The inset shows the current-time response during polarization at 10 mV.

**Figure S15.** EIS spectra of ZnSO_4_ and F-ZnSO_4_ (left and right respectively) at different temperatures.

**Figure S16.** Arrhenius curves and comparison of activation energies in F-ZnSO_4_ and ZnSO_4_.

**Figure** **S17.** Schematic of the growth orientation of the Zn (002) and Zn (100) crystal planes.

**Figure S18.** Grazing-incidence X-ray diffraction (GI-XRD) patterns of the Zn anodes after 20, and 100 cycles in the F-ZnSO_4_ electrolyte.

**Figure S19.** SEM characterization of the Zn anodes cycled in the F-ZnSO_4_ electrolyte: (a, b, c, d) after 100 cycles.

**Figure S20.** XPS spectra for C 1s of the surface of Zn foil after 10 times cycled in F-ZnSO_4_ (top) and Zn foil cycled in ZnSO_4_ (bottom).

**Figure S21.** XPS spectra for F 1s of the surface of Zn foil in F-ZnSO_4_.

**Figure S22.** In situ optical microscopy image of Zn-electrolyte interface during Zn deposition in ZnSO_4_ and F-ZnSO_4_. The current density was 10 mA cm^-2^.

**Figure S23.** SEM images of the Zn foils cycled with ZnSO_4_ (top)and F- ZnSO_4_ (bottom) electrolytes after 20 cycles at 2 mA cm^-2^.

**Figure S24.** Galvanostatic cycling curves of Zn||Zn symmetric cells at 2 mA cm^-2^, with a fixed capacity of 1mAh cm^-2^.

**Figure S25.** Rate performances of Zn||Zn symmetric cells at different current densities with a fixed capacity of 1 mAh cm^-2^.

**Figure S26.** CV curves of the Zn||MnO_2_ full cell with the scan rate of 0.2 mV s^-1^ to 1 mV s^-1^.

**Figure S27.** Cycling properties of Zn||MnO_2_ full cells at 0.2 A g^-1^.

**Supplementary Notes**

**1. Extended computational analysis of interfacial fluorinated ion crowding**

To further clarify the interfacial fluorinated ion-crowding mechanism, additional computational analyses were performed to examine ion adsorption, surface-binding configurations, bulk solvation characteristics, and electrostatic features near the Zn surface.

The interfacial affinities of representative ionic species on different Zn crystal planes were first evaluated. As shown in Supplementary Fig. 28, both SO_4_^2−^ and Zn^2+^ exhibit clear facet-dependent adsorption energies, indicating that ion-surface interactions are strongly influenced by the crystallographic nature of the Zn substrate. Optimized adsorption configurations of BF_4_^−^, OTf^−^, TFSI^−^, SO_4_^2−^, and Zn^2+^ on different Zn surfaces are presented in Supplementary Figs. 29-33. These results show distinct surface-binding geometries for different species, supporting the view that the interfacial region is governed by species-dependent adsorption and spatial organization rather than random ion accumulation.

To provide a reference for the bulk electrolyte environment, the radial distribution function and coordination number of Zn in 2M ZnSO_4_ were further analysed (Supplementary Fig. 34). The obtained solvation characteristics are consistent with a preserved bulk Zn^2+^ solvation structure, supporting the conclusion that the fluorinated additives mainly regulate the interfacial region while introducing only limited perturbation to bulk solvation.

The corresponding electric-field and electrostatic-potential profiles are shown in Supplementary Figs. 35 and 36. Both quantities decay rapidly away from the Zn surface and converge in the bulk electrolyte region, consistent with the expected behavior of a stabilized electrical double layer. This bulk-referenced electrostatic profile further supports the physical validity of the simulated interfacial structure.

Overall, the extended computational results support a mechanism in which fluorinated anions with distinct adsorption characteristics cooperatively accumulate near the Zn surface, suppress interfacial water access, and promote local Zn^2+^ enrichment, thereby regulating Zn deposition through an interfacially confined ion-crowding effect rather than a bulk-solvation-dominated pathway.

**Figure S28.** The adsorption energy of SO_4_^2-^ and Zn^2+^ on different surfaces of Zn.

**Figure S29.** The optimized structure when BF_4_^-^ was adsorbed on different surfaces of Zn. The corresponding Zn^2+^ and BF_4_^-^ ion is omitted for clarity.

**Figure S****30.** The optimized structure when OTF^-^ was adsorbed on different surfaces of Zn. The corresponding Zn^2+^ and OTF^-^ ion is omitted for clarity.

**Figure S****31.** The optimized structure when TFSI^-^ was adsorbed on different surfaces of Zn. The corresponding Zn^2+^ and TFSI^-^ ion is omitted for clarity.

**Figure S****32.** The optimized structure when SO_4_^2-^ was adsorbed on different surfaces of Zn. The corresponding Zn^2+^ ion is omitted for clarity.

**Figure S****33.** The optimized structure when Zn^2+^ was adsorbed on different surfaces of Zn. The corresponding SO_4_^2-^ ion is omitted for clarity.

**Figure.S34** The radial distribution function and the coordination number of Zn in 2M ZnSO_4_ solution.

**Figure S35.** The change of the electric field with the distance from surface.

**Figure S36.** The change of the electric potential with the distance from surface.

**2. Additional characterization of the fluorinated interphase during immersion and cycling**

To further characterize the evolution of the fluorinated interphase, additional ex situ X-ray photoelectron spectroscopy (XPS) and Raman measurements were performed on Zn electrodes in F-ZnSO_4_ electrolytes under different conditions.

As shown in Supplementary Fig. 37, the Zn electrode after 10 cycles exhibits clear F, S, O, Zn, B, C, and N signals. In particular, the F 1s spectrum is dominated by a peak at ~685 eV, consistent with Zn-F bonding, indicating the formation of a fluorine-containing interfacial layer after electrochemical cycling.

After immersion in F-ZnSO_4_ for 24 h, similar elemental signatures remain observable (Supplementary Fig. 38). The corresponding F 1s spectrum shows two components at ~684.7 eV and ~688 eV, suggesting the coexistence of Zn-F species and higher-binding-energy fluorinated species under static conditions. Compared with the immersed sample, the cycled interface is therefore chemically more well-defined and enriched in stable Zn-F bonding environments.

Raman spectra collected from Zn anodes deposited for 30 s, 3 min, and 9 min are shown in Supplementary Fig. 39. The O-H stretching bands decrease progressively with deposition time, indicating gradual depletion of interfacial water during Zn growth. This trend is consistent with the accumulation of fluorinated species at the interface and the reduced participation of water in interfacial side reactions.

**Figure S37.** XPS analysis of the Zn foil after 10 cycles in the F-ZnSO_4_ electrolyte, including the spectra of F, S, O, Zn, B, C, and N elements.

**Figure S38.** XPS analysis of the Zn foil after immersion in the F-ZnSO_4_ electrolyte for 24 hours, including the spectra of F, S, O, Zn, B, C, and N elements.

**Figure S39.** Raman analysis of Zn anodes deposited for 30 s, 3 min, and 9 min in the F-ZnSO_4_ electrolyte.

**3. Post-mortem structural analysis of Zn and MnO_2_ electrodes in Zn||MnO_2_ full cells**

To further evaluate the structural reversibility of both electrodes, post-mortem X-ray diffraction (XRD) analyses were performed on the Zn anode and MnO_2_ cathode after 2 cycles in Zn||MnO_2_ cells.

As shown in Supplementary Fig. S40, In the ZnSO_4_ electrolyte, anode exhibit markedly stronger by-product signals, including basic zinc sulfate species (ZHS) on the Zn surface derived from the local accumulation of OH⁻ generated by irreversible HER, and electrodes cycled in the F-ZnSO_4_ electrolyte present notably “cleaner” XRD patterns, with substantially weaker by-product peaks.

The cycled MnO_2_ cathodes were further examined. As shown in Supplementary Fig. S41, In the ZnSO_4_ electrolyte, Zn-Mn secondary phases on the MnO_2_ cathode originating from irreversible H⁺ insertion that leaves excess OH⁻ to react with MnO_2_. The cathode from F-ZnSO_4_ retains a cleaner phase composition with fewer parasitic products.

These results indicate that the fluorinated electrolyte effectively suppresses parasitic structural evolution on both the Zn anode and MnO_2_ cathode, thereby contributing to the enhanced reversibility of Zn||MnO_2_ full cells.

**Figure S40.** Post-mortem XRD patterns of the Zn anode after 2 cycles in Zn||MnO_2_ full cells.

**Figure S41.** Post-mortem XRD patterns of the MnO_2_ Cathode after 2 cycles in Zn||MnO_2_ full cells using ZnSO_4_ and F-ZnSO_4_ electrolytes

**Table S1.** Zeta potential of the ZnSO_4_ and F-ZnSO_4_ electrolytes.

| Sample | Measurement No. | \|Zeta Potential\| (mV) |
| --- | --- | --- |
| ZnSO_4_ | 1 2 | 5.53 4.11 |
|  | 3 | 4.17 |
|  | 1 | 19.4 |
| F-ZnSO_4_ | 2 | 19.7 |
|  | 3 | 20.6 |

The average zeta potentials calculated from three measurements are 4.6 mV for ZnSO_4_ and 19.9 mV for F-ZnSO_4_, respectively.

**Table S2.** Calculation of ionic conductivity (*σ*) for two electrolyte systems

| **Parameter (Symbol)** | **F-ZnSO₄** | **ZnSO₄** |
| --- | --- | --- |
| Electrode diameter (*D*) | 1.2 cm | 1.2 cm |
| Electrode radius (*r*) | 0.6 cm | 0.6 cm |
| Electrode area (*A* = π*r*^2^) | 1.131 cm^2^ | 1.131 cm^2^ |
| Electrode spacing (*d*) | 0.052 cm | 0.052 cm |
| Resistance (R) | 1.58 Ω | 1.67 Ω |
| Ionic conductivity (*σ* = *d*/ (R × *A*)) | **0.0291 S·cm^-1^** | **0.0275 S·cm^-1^** |

**Table S3**. Comparison of the performance of some recent works on Zn||MnO_2_ batteries.

| Anode Material | Cathode Material | Electrolyte Material | | Capacity | References | |
| --- | --- | --- | --- | --- | --- | --- |
| Zn powder-Zn foil | MnO_2_ powder-carbon paper | 2 M ZnSO_4_ + 0.1 M MnSO_4_  aqueous electrolyte | | 233 mAh·g^-1^ (under 1 C, based on the mass of MnO_2_) | Energy Storage Materials, 2018, 15, 351. | |
| Zn foil | α-MnO_2_ electrodes | 1 M Zn(OTf)_2_/DMF + 2 vol% H_2_O electrolyte | | 91.1 mAh g^-1^, (at 1.0 A g^-1^) | Angewandte Chemie:2025, e15055. | |
| zinc foil | H-MnO_2_ | 2 M ZnSO_4_ aqueous electrolyte | | Around 100 mAh g^−1^(at 0.4A g^-1^ | Advanced Functional Materials 34(42): 2405659. | |
| Zinc foil | δ-MnO_2_ powder-carbon paper | 1 M Zn(TFSI)_2_+0.1 M Mn(TFSI)_2_ aqueous  electrolyte | | 136.9 mAh·g^-1^ (under 20 C, based on the mass of MnO_2_) | Advanced Materials, 2019, 31, 1900567. | |
| Zinc foil | nanofiber α-MnO_2_-carbon sheet | 2 M ZnSO_4_+0.1 M MnSO_4_ aqueous electrolyte | 285 mAh·g^-1^ (under 1/3 C, based on the mass of MnO_2_) | | Nature Energy, 2016, 1, 16039. |  |
| MOF coated Zn | MnO_2_ | 2 M ZnSO_4_ aqueous electrolyte | Around 150 mAh g^−1^at 0.7A g^-1^ | | Angewandte Chemie 132(24): 9463-9467. |  |
| Polished Zinc foils | Commercial MnO_2_ | 2 M ZnSO_4_ + 0.1 M MnSO_4_ + 1 mM NH_3_·H_2_O | 97 mAh g^-1^, (at 1.0 A g^-1^) | | Nano-Micro Letters 15: 81. |  |
| Zn foil | Commercial MnO_2_ | 1 M ZnSO_4_+0.1 M MnSO_4_+25 mM NHP | Around 100 mAh g^-1^, (at 1.0 A g^-1^) | | Angewandte Chemie 62(5): e202212695. |  |
| Zn foil | Commercial MnO_2_ | 2 M ZnSO_4_ +5 mM Zn(CF_3_SO_3_)_2_ +5 mM Zn(BF_4_)_2_·*n*H_2_O +5 mM zinc di[bis(trifluoromethylsulfonyl)imide] | 105 mAh g^-1^, (at 1.0 A g^-1^) | | This Study |  |

**Table S4.** Ionic radii of the TFSI⁻, OTf⁻, and BF_4_⁻ anions

| **Anion** | **Ion Radius** |
| --- | --- |
| TFSI^-^ | 0.326 nm |
| OTF^-^ | 0.267 nm |
| BF_4_^-^ | 0.227 nm |

**References**

(1) Thompson, A. P.; Aktulga, H. M.; Berger, R.; Bolintineanu, D. S.; Brown, W. M.; Crozier, P. S.; in 't Veld, P. J.; Kohlmeyer, A.; Moore, S. G.; Nguyen, T. D.; et al. LAMMPS - a flexible simulation tool for particle-based materials modeling at the atomic, meso, and continuum scales. *Comput. Phys. Commun.* 2022, *271*.

(2) (a) Gingrich, T. R.; Wilson, M. On the Ewald summation of Gaussian charges for the simulation of metallic surfaces. *Chem. Phys. Lett.* 2010, *500* (1-3), 178-183. (b) Reed, S. K.; Lanning, O. J.; Madden, P. A. Electrochemical interface between an ionic liquid and a model metallic electrode. *J. Chem. Phys.* 2007, *126* (8), 084704. (c) Siepmann, J. I.; Sprik, M. Influence of surface topology and electrostatic potential on water/electrode systems. *J. Chem. Phys.* 1995, *102* (1), 511-524. (d) Wang, Z.; Yang, Y.; Olmsted, D. L.; Asta, M.; Laird, B. B. Evaluation of the constant potential method in simulating electric double-layer capacitors. *J. Chem. Phys.* 2014, *141* (18), 184102.

(3) Ahrens-Iwers, L. J. V.; Janssen, M.; Tee, S. R.; Meissner, R. H. ELECTRODE: An electrochemistry package for atomistic simulations. *J. Chem. Phys.* 2022, *157* (8), 084801.

(4) Price, D. J.; Brooks, C. L., 3rd. A modified TIP3P water potential for simulation with Ewald summation. *J. Chem. Phys.* 2004, *121* (20), 10096-10103.

(5) Miyamoto, S.; Kollman, P. A. Settle: An analytical version of the SHAKE and RATTLE algorithm for rigid water models. *J. Comput. Chem.* 2004, *13* (8), 952-962.

(6) (a) Cauet, E.; Bogatko, S.; Weare, J. H.; Fulton, J. L.; Schenter, G. K.; Bylaska, E. J. Structure and dynamics of the hydration shells of the Zn^2+^ ion from ab initio molecular dynamics and combined ab initio and classical molecular dynamics simulations. *J. Chem. Phys.* **2010**, *132* (19), 194502. (b) Liu, Z.; Huang, S.; Wang, W. A Refined Force Field for Molecular Simulation of Imidazolium-Based Ionic Liquids. *J. Phys. Chem. B* 2004, *108* (34), 12978-12989. (c) Ȧqvist, J. Ion-water interaction potentials derived from free energy perturbation simulations. *The Journal of Physical Chemistry* 1990, *94* (21), 8021-8024. (d) Canongia Lopes, J. N.; Pádua, A. A. H. Molecular Force Field for Ionic Liquids Composed of Triflate or Bistriflylimide Anions. *J. Phys. Chem. B* 2004, *108* (43), 16893-16898.

(7) Evans, D. J.; Holian, B. L. The Nose–Hoover thermostat. *J. Chem. Phys.* **1985**, *83* (8), 4069-4074.
